# Smartphone use among nursing students during clinical placements: a five-year comparison of use, distraction, and regulatory perceptions

**DOI:** 10.3389/fpubh.2026.1987602

**Published:** 2026-10-08

**Authors:** Sergio Yuste, Jagoba Zarandona, Susana Cariñanos-Ayala, Isabel De Jesus Oliveira, Itziar Hoyos Cillero

**Affiliations:** 1 Basque Nurse Education Research Group, Bioaraba Health Research Institute, Vitoria-Gasteiz, Spain; 2 Nursing School, Osakidetza-Basque Health Service, Vitoria-Gasteiz, Spain; 3 Health Sciences Research Unit: Nursing, Nursing School of Coimbra, Univ Coimbra, Coimbra, Portugal; 4 Faculty of Medicine and Nursing, Department of Nursing, University of the Basque Country (UPV/EHU), Leioa, Spain

**Keywords:** distraction, nursing education, patient safety, preceptorship, smartphone

## Abstract

**Aim:**

To identify patterns of smartphone use among nursing students during clinical placements, examine perceptions of smartphone use as a professional tool and a source of distraction, explore attitudes towards smartphone use policies, and compare findings with data collected 5 years earlier.

**Methods:**

A descriptive cross-sectional study compared two independent cohorts of nursing students from a Spanish nursing school: Period I (2016/17; *n* = 234) and Period II (2023/24; *n* = 203). Data were collected using the same structured questionnaire, assessing smartphone use, distraction experiences, and attitudes towards restriction policies. Descriptive and non-parametric analyses were performed.

**Results:**

Professional and academic smartphone use increased significantly over the five-year period, particularly for communication with healthcare professionals, access to clinical information, use of healthcare applications and academic tasks. Social media use remained highly prevalent in both cohorts. Despite greater smartphone use, self-reported distraction did not increase, while distraction caused by peers decreased. Students in Period II expressed less support for restrictive smartphone policies, particularly in hospital settings. Professional and academic smartphone use were positively associated with perceived work efficiency and less restrictive attitudes towards smartphone use.

**Conclusion:**

Smartphones have become increasingly integrated into nursing students’ clinical learning experiences. Greater professional and academic use was not accompanied by increased perceived distraction, suggesting an ongoing normalization of smartphones as educational and clinical tools. The findings support promoting digital professionalism and responsible smartphone use rather than relying primarily on restrictive policies.

## 1 Introduction

The manner in which healthcare professionals access information, communicate, and provide care has undergone a profound transformation as a consequence of the integration of technology into the healthcare sector (1, 2). This transformation has been largely driven by the accessibility, portability, and real-time information capabilities of digital tools, particularly mobile devices (3).

As part of this digital transformation, mobile technologies have emerged as widely used tools among students in nursing, medicine and other health-related disciplines (4–6). Over the past decade, training in healthcare professions has gradually moved away from traditional classroom-based approaches towards blended, technology-enhanced models that use mobile devices for learning and clinical support (4, 7). During periods of clinical placement, students have the capacity to access a wide variety of resources via their smartphones. These resources encompass clinical guidelines, pharmacological resources, scientific databases, educational applications and decision-support tools (3, 8). Such accessibility reinforces the role of smartphones as key tools within the clinical learning environment.

Building on this recognition, health and education institutions have emphasised the need to address the issue of smartphone usage among nursing students (3). The aim is to find an appropriate balance between the educational advantages offered by these devices and the potential hazards that accompany them, such as distraction, breaches of professional conduct and risks to patient safety (9, 10).

The existing body of research is predominantly focused on the description of students’ perceptions, frequency of use and concerns about distraction (1, 11, 12). However, there is still limited evidence regarding how these patterns evolve over time, particularly in relation to changes in students’ behaviours, perceptions of distraction, and attitudes towards institutional regulations governing smartphone use during clinical placements. Furthermore, few studies have comparatively examined these aspects within the same institutional context.

Studies involving nursing students indicate that mobile devices are perceived as useful tools that facilitate learning, improve access to updated knowledge and assist students when facing uncertainty in clinical environments (3, 8, 13). Nevertheless, the integration of smartphones into healthcare settings also gives rise to new challenges. While these tools can serve a professional function, their inappropriate use can result in disruptions to patient care, particularly when they are used for personal activities such as messaging, social media or web browsing that are unrelated to clinical practice (1, 12, 14). This coexistence of educational potential and risk highlights the complexity of smartphone use in clinical training environments.

This dual nature of smartphone use, simultaneously a clinical resource and a potential source of distraction, underscores the need to better understand the trends shaping their use among nursing students, including their perception of smartphones as a distraction and their views on policies that regulate their use during clinical placements (10, 11, 13).

In this context, understanding how nursing students use smartphones during clinical placements, how they perceive their potential both as a source of distraction and as a tool for professional use and how they evaluate existing regulations becomes essential for informing educational strategies and policy development. Moreover, examining changes over time can provide valuable insights into the evolution of these behaviours in response to increasing digitalization.

The aims of this study were to: (1) identify patterns of smartphone use among nursing students during clinical placements, with particular attention to professional applications; (2) assess students’ perceptions of smartphones as a source of distraction; (3) examine students’ views on regulations governing smartphone use in clinical settings; and (4) compare these findings with data collected 5 years earlier.

## 2 Methods

### 2.1 Design

A descriptive cross-sectional study design was used. This study was conducted and reported in accordance with the STROBE guidelines for cross-sectional studies (15).

### 2.2 Participants

To meet the objectives of this study, all nursing students enrolled during the 2023/24 academic year were eligible to participate, provided that they had completed at least one clinical placement. Students who had not undertaken any clinical placement during the 2023/24 academic year were excluded from the study.

Using a non-probabilistic convenience sampling strategy, nursing students were recruited from a public school of nursing in Spain. The study compares two independent cohorts collected 5 years apart. In Period I (2016/17), 234 students participated from a total population of 303 eligible students (participation rate: 77.2%). In Period II (2023/24), 203 students participated from a total population of 340 eligible students (participation rate: 59.7%). The relatively high participation rates in both periods provided broad coverage of the target populations and strengthened the robustness of the findings.

### 2.3 Instrument

The structured questionnaire originally used in Period I *[reference 1 anonymized for review]* was retained for Period II to facilitate comparisons between the two study periods. The questionnaire consists of five sections with a total of 38 items. The first section collects sociodemographic and educational data, including age, gender, academic year, and location of the most recent clinical placement. The second section includes seven items examining general patterns of digital device use in daily life. The third section comprises 14 items assessing the frequency of professional smartphone use during clinical placements. The fourth section contains nine items focused on smartphone-related distraction in clinical settings, while the fifth section includes four items exploring students’ perceptions of policies regulating smartphone use. Items related to professional use and distraction are measured using a 5-point Likert scale ranging from never (1) to always (>10 times per day) (5). Similarly, items assessing attitudes towards smartphone use policies (as well as Question 10) are measured using a 5-point Likert scale ranging from strongly disagree (1) to strongly agree (5).

Internal consistency was assessed for the third, fourth, and fifth sections of the questionnaire using Cronbach’s alpha coefficients. Cronbach’s alpha values were 0.678 in the Period I sample and 0.765 in the Period II sample, indicating moderate internal consistency in Period I and acceptable internal consistency in Period II. Sociodemographic characteristics and smartphone use-related variables were not included in the reliability analysis, as they were considered descriptive variables rather than scale-based measures.

### 2.4 Data collection

Data were collected during two study periods separated by 5 years. In Period I (2016/17 academic year), data collection took place between March and June 2017. At the end of scheduled teaching activities associated with clinical placements, the study was presented to students by the principal investigator, who explained the study objectives and emphasized the voluntary nature of participation. Paper-based questionnaires were distributed in class and students were given one week to complete and return them anonymously to a collection box located at the School of Nursing. To minimize potential pressure to participate, no members of the teaching staff or research team remained present during questionnaire completion.

In Period II (2023/24 academic year), data collection was conducted between January and June 2024, coinciding with the completion of clinical placement rotations and scheduled on-campus teaching sessions. Before the start of a lecture, a member of the research team introduced the study, explained its objectives, and answered any questions. Students were then invited to participate voluntarily and those who agreed received a link to an online questionnaire administered through Microsoft® Forms. The survey was self-administered and completed electronically, allowing standardized data collection across participants.

During both periods, data collection focused on gathering quantitative information about students’ smartphone use during clinical placements and the purposes for which smartphones were used. The study did not assess the quality, validity, currency, or reliability of the information or digital resources accessed.

### 2.5 Data analysis

Analysis was performed using IBM SPSS® Statistics for Windows, Version 29.0. The Kolmogorov–Smirnov test was applied to assess the normality of continuous variables. Descriptive statistics, including frequencies, total percentages, averages, and standard deviations, were employed to examine the general characteristics of nursing students, the frequency of smartphone usage, experiences of distraction, and views on smartphone restriction policies.

Given the non-normal distribution of the variables, non-parametric statistical tests were applied. The Mann–Whitney U test was conducted to compare experiences of distraction and opinions on smartphone restriction policies across different clinical practice locations. Additionally, the Chi-square and Kruskal-Wallis tests were used to investigate differences in mobile use for personal reasons and distractions caused by others’ mobile use among various academic years. For longitudinal comparisons between Period I and Period II, Chi-square and Mann–Whitney U tests were applied depending on the type of variable.

Spearman’s rho correlation analysis was used to explore the relationships between different dimensions of distraction (own use and others’ use) and students’ attitudes towards smartphone restriction policies, professional use of smartphones, academic use of smartphones, and perceived work efficiency when using smartphones. The significance level was established at less than 0.05.

### 2.6 Ethics

This study was approved by the Dean of the School of Nursing. Participants were informed that completion of the questionnaire implied their consent to participate. The Ethics Committee for research involving human subjects, their samples and data of the University of the Basque Country UPV/EHU reviewed and approved this research project *(anonymized for review)*.

## 3 Results

A total of 234 participants were included in Period I and 203 in Period II. Sociodemographic characteristics, daily smartphone use, and patterns of smartphone use are summarized in Table 1.

**Table 1:** Participants’ sociodemographic characteristics for Period I and Period II.

Variable		Period I	Period II	*p* ^a^
		*N* = 234 (%)	*N* = 203 (%)	
Sex	Man	27 (11.5)	40 (19.7)	0.032
	Woman	202 (86.3)	163 (80.3)	
	Non-binary	—	—	
Age, years	18–25	210 (91.7)	184 (92.5)	0.426
	26–30	13 (5.7)	7 (3.5)	
	31–50	6 (2.6)	8 (4)	
Practicum year	Introduction to practicum (1st year)	35 (15.0)	34 (16.7)	0.631
	Practicum I (2nd year)	71 (30.3)	70 (34.5)	
	Practicum III (3rd year)	71 (30.3)	52 (25.6)	
	Practicum V (4th year)	57 (24.4)	47 (23.2)	
Most recent clinical placement	Hospital	150 (64.1)	155 (76.7)	0.004
	Non-hospital	84 (35.9)	47 (23.3)	
Type of mobile phone	Smartphone	232 (99.1)	203 (100)	0.187
	Non-smartphone	2 (0.9)	—	
	None	—	—	
Amount of smartphone use	< 1 h/day	10 (4.3)	4 (2)	0.009
	1–3 h/day	82 (35.3)	59 (29.1)	
	3–5 h/day	92 (39.7)	112 (55.2)	
	>5 h/day	48 (20.7)	28 (13.8)	
Reason for smartphone use	Social networks	227 (98.3)	188 (92.6)	0.004
	WhatsApp	223 (96.5)	200 (98.5)	0.189
	Instagram	157 (68)	188 (92.6)	<0.001
	Facebook	142 (61.5)	9 (4.4)	<0.001
	YouTube	127 (55)	91 (44.8)	0.035
	Twitter	56 (24.2)	76 (37.4)	0.003
	Snapchat	45(19.5)	—	—
	Tik Tok	—	113 (55.7)	—
	Surfing the internet	180 (77.9)	121 (59.9)	<0.001
	Sending/receiving messages	67 (29)	148 (72.9)	<0.001
	Voice calls	155 (67.1)	99 (48.8)	<0.001
	Music and videos	165 (71.4)	164 (80.8)	0.023
	Games	27 (11.7)	21 (10.3)	0.656

PI Period 1; PII Period 2.

^a^Chi-square statistics.

The proportion of men was significantly higher in Period II than in Period I (19.7% vs. 11.5%, *p* = 0.032), although most participants were women in both periods (80.3% vs. 86.3%). Age distribution did not differ significantly, with the majority of participants aged 18–25 years in both periods (92.5% in Period II vs. 91.7% in Period I). No significant differences were observed in academic year distribution, with a comparable distribution across years. However, hospital-based clinical placements were more frequent in Period II than in Period I (76.7% vs. 64.1%, *p* = 0.004).

Smartphone ownership was nearly universal in both periods (≥99%). In contrast, daily smartphone use differed significantly (*p* = 0.009): participants in Period II more frequently reported using smartphones for 3–5 h per day (55.2% vs. 39.7%), whereas use of more than 5 h per day was more common in Period I (20.7% vs. 13.8%).

Social network use was highly prevalent in both periods, although slightly lower in Period II compared to Period I (92.6% vs. 98.3%, *p* = 0.004). Instagram and Twitter use increased in Period II compared to Period I (92.6% vs. 68.0%, *p* < 0.001; 37.4% vs. 24.2%, *p* = 0.003, respectively), while Facebook use declined markedly (4.4% vs. 61.5%, *p* < 0.001). YouTube use decreased modestly (44.8% vs. 55.0%, *p* = 0.035), whereas WhatsApp remained consistently high (>96%) in both periods. Snapchat was reported only in Period I (19.5%), while TikTok use emerged as a highly prevalent platform in Period II (55.7%).

Other uses of smartphones also changed significantly between periods. Internet browsing and voice calls decreased (59.9% vs. 77.9 and 48.8% vs. 67.1%, respectively; both *p* < 0.001), whereas messaging (72.9% vs. 29.0%, *p* < 0.001) and use for music and video consumption (80.8% vs. 71.4%, *p* = 0.023) were more frequently reported in Period II compared to Period I. No significant differences were observed in gaming.

### 3.1 Frequency and patterns of professional smartphone use during practicum

Table 2 shows the frequency and professional use of smartphones during practicum in Period I and Period II. Significant differences between periods were observed for most professional use items.

**Table 2:** Frequency of use and professional use of the smartphone during practicum for Period I and Period II.

Items		Frequency *N* (%)							
		Never	Rarely	Sometimes	Often	Always	*p* ^a^	Mean (SD)	*p* ^b^
Q1-I use my mobile as a tool for communicating and coordinating with other members of the team.	PI	141 (60.8)	45 (19.4)	22 (9.5)	17 (7.3)	6 (2.6)	<0.001	1.70 (1.078)	0.037
	PII	91 (44.8)	80 (39.4)	26 (12.8)	6 (3)	—		1.74 (0.793)	
Q2-I access the Internet to find answers to queries about drugs.	PI	58 (25.2)	58 (25.2)	68 (29.6)	35 (15.2)	11 (4.8)	<0.001	2.49 (1.163)	0.069
	PII	21 (10.3)	63 (31)	87 (42.9)	31 (15.3)	1 (0.5)		2.65 (0.88)	
Q3-I access apps that help me with patient care.	PI	163 (70.9)	35 (15.2)	21 (9.1)	7 (3)	3 (1.3)	<0.001	1.47 (0.885)	<0.001
	PII	98 (48.3)	67 (33)	29 (14.3)	7 (3.4)	2 (1)		1.76 (0.893)	
Q4-I use my mobile as a calculator for medication doses.	PI	105 (45.3)	36 (15.5)	46 (19.8)	33 (14.2)	12 (5.2)	<0.001	2.19 (1.288)	0.059
	PII	54 (26.6)	71 (35)	43 (21.2)	27 (13.3)	8 (3.9)		2.33 (1.123)	
Q5-I surf the Internet to search for information related to the clinical placement.	PI	92 (39.8)	48 (20.8)	62 (26.8)	20 (8.7)	6 (2.6)	0.011	2.10 (1.138)	0.019
	PII	58 (28.6)	54 (26.6)	60 (29.6)	30 (14.8)	1 (0.5)		2.32 (1.058)	
Q6-I access university resources.	PI	80 (34.5)	54 (23.3)	50 (21.6)	36 (15.5)	12 (5.2)	0.117	2.34 (1.241)	0.548
	PII	63 (31)	63 (31)	51 (25.1)	19 (9.4)	7 (3.4)		2.23 (1.095)	
Q7-I submit documents for academic assessment	PI	176 (76.2)	27 (11.7)	11 (4.8)	9 (3.9)	8 (3.5)	<0.001	1.47 (0.999)	<0.001
	PII	98 (48.3)	49 (24.1)	29 (14.3)	13 (6.4)	13 (6.4)		1.98 (1.213)	
Q8-I use my mobile to send messages/emails to colleagues containing patient data (e.g., their reference number, bed number, medical record number, etc.)	PI	218 (94.4)	9 (3.9)	2 (0.9)	2 (0.9)	—	0.276	1.08 (0.381)	0.167
	PII	197 (97)	6 (3)	—	—	—		1.03 (0.17)	
Q9-I have seen my tutor/reference nurse use their personal mobile to send messages/emails to colleagues containing patient data	PI	200 (86.2)	15 (6.5)	9 (3.9)	4 (1.7)	4 (1.7)	0.218	1.26 (0.764)	0.466
	PII	169 (83.3)	20 (9.9)	11 (5.4)	3 (1.5)	—		1.25 (0.622)	

Items		Strongly disagree	Disagree	Uncertain	Agree	Strongly agree	*p* ^a^	Mean (SD)	*p* ^b^
Q10-My use of my smartphone makes my work more efficient	PI	19 (8.2)	54 (23.4)	93 (40.3)	56 (24.2)	9 (3.9)	0.241	2.92 (0.979)	0.264
	PII	10 (5)	38 (18.8)	99 (49)	44 (21.8)	11 (5.4)		3.04 (0.908)	

Items	Duration of use	PI *N* (%)	PII *N* (%)	*p* ^a^
PI-Q11-In working hours during my practicum I use my mobile	<1 h/day	217 (94.8)	—	0.006
	1–3 h/day	12 (5.2)	202 (99.5)	
	3–5 h/day	—	1 (0.5)	
	>5 h/day	—	—	
	3–5 h/day	—	—	

PI Period 1, PII Period 2, SD standard deviation.

^a^ Chi-square statistics.

^b^ Mann–Whitney U statistics.

Smartphone use for communication and coordination with team members (Q1) increased in Period II, with responses shifting from “never” being the most frequent category in Period I (60.8%) to higher proportions of rare and occasional use in Period II (*p* < 0.001). Similarly, accessing the internet for drug-related queries (Q2), using apps for patient care (Q3), using the mobile as a medication dose calculator (Q4), and searching for clinical information during placements (Q5) all showed significant differences between periods (all *p* < 0.001), with generally higher use reported in Period II. Across Q2–Q4, responses shifted from “never” being the most frequent category in Period I to higher proportions of rare, occasional, and frequent use in Period II, with a similar trend observed for Q5.

Mean values were significantly higher in Period II for Q1 (*p* = 0.037), Q3 (*p* < 0.001), and Q5 (*p* = 0.019), indicating an overall increase in professional smartphone use during clinical placements.

Use of smartphones for academic purposes also changed. Submitting documents for academic assessment (Q7) significantly increased in Period II (*p* < 0.001), with responses shifting from “never” being the most frequent category in Period I to higher proportions of rare, occasional, frequent, and very frequent use in Period II. Mean values were also significantly higher in Period II (*p* < 0.001), reflecting increased academic use.

In contrast, accessing university resources (Q6) and reporting of potentially unprofessional or unsafe communication practices (Q8 and Q9) did not show significant differences between periods, either in frequency distributions or in mean scores.

Regarding perceived efficiency, most students reported neutral to positive perceptions of the impact of smartphone use on work efficiency (Q10), with similar mean scores and no significant differences between periods.

Finally, smartphone use during working hours (Q11) showed a significant shift in distribution (*p* = 0.006), with most students in Period II reporting 1–3 h of smartphone use per day during practicum.

### 3.2 Frequency of smartphone use for personal purposes, associated distraction, and opinions on restriction policies during clinical practice

Table 3 shows the frequency of smartphone use for personal purposes, associated distraction, and opinions on restriction policies in Period I and Period II.

**Table 3:** Frequency of smartphone use and resultant distraction, and opinion about restriction policies for Period I and Period II.

Item		Frequency *N* (%)							
		Never	Rarely	Sometimes	Often	Always	*p* ^a^	Mean (SD)	*p* ^b^
Q12-I have used my mobile for personal matters (e.g., calling, sending messages, accessing social networks, etc.) during the practicum	PI	96 (41.4)	82 (35.3)	38 (16.4)	13 (5.6)	3 (1.3)	0.049	1.90 (0.955)	0.937
	PII	73 (36)	93 (45.8)	33 (16.3)	3 (1.5)	1 (0.5)		1.85 (0.778)	
Q13-I’ve answered/made phone calls and/or sent messages or emails from my mobile when I was with a patient	PI	211 (93)	9 (4)	3 (1.3)	2 (0.9)	1 (0.4)	0.860	1.11 (0.486)	0.896
	PII	191 (94.1)	8 (3.9)	2 (1)	2 (1)	—		1.09 (0.401)	
Q14-I’ve answered/made phone calls and/or sent messages or emails from my mobile when I was in a training session.	PI	179 (77.2)	34 (14.7)	15 (6.5)	2 (0.9)	2 (0.9)	0.297	1.34 (0.714)	0.283
	PII	162 (80.6)	32 (15.9)	6 (3)	1 (0.5)	—		1.23 (0.52)	
Q15-Have you been distracted by your use of your smartphone during your practicum?	PI	176 (75.9)	40 (17.2)	16 (6.9)	—	—	0.108	1.31 (0.595)	0.656
	PII	156 (76.8)	41 (20.2)	5 (2.5)	1 (0.5)	—		1.27 (0.525)	
Q16-Have you been distracted by other students’ use of their smartphone during your practicum?	PI	159 (68.8)	44 (19)	24 (10.4)	3 (1.3)	—	0.034	1.43 (0.736)	0.029
	PII	158 (77.8)	36 (17.7)	8 (3.9)	—	1 (0.5)		1.28 (0.583)	
Witnessing other students’ and nurses’ smartphone use and distraction									
Q17-Have you seen other students’ being distracted by the use of their smartphone during the practicum?	PI	132 (57.1)	49 (21.2)	44 (19)	5 (2.2)	1 (0.4)	0.434	1.68 (0.886)	0.731
	PII	115 (56.7)	55 (27.1)	27 (13.3)	5 (2.5)	1 (0.5)		1.63 (0.842)	
Q18-Have you seen nurses being distracted by the use of their smartphone during their working hours?	PI	73 (31.5)	70 (30.2)	63 (27.2)	20 (8.6)	6 (2.6)	0.871	2.21 (1.061)	0.828
	PII	62 (30.5)	62 (30.5)	54 (26.6)	22 (10.8)	3 (1.5)		2.22 (1.046)	
Q19-I have seen my tutor/reference nurse answer/make phone calls and/or send messages or emails when he/she was with a patient.	PI	146 (62.9)	37 (15.9)	27 (11.6)	15 (6.5)	7 (3)	0.051	1.71 (1.093)	0.348
	PII	116 (57.4)	43 (21.3)	19 (9.4)	23 (11.4)	1 (0.5)		1.76 (1.057)	
Q20-I have seen my tutor/reference nurse answer/make phone calls and/or send messages or emails when he/she was in a training session.	PI	134 (57.8)	50 (21.6)	26 (11.2)	15 (6.5)	6 (2.6)	0.127	1.73 (1.064)	0.634
	PII	112 (55.2)	51 (25.1)	20 (9.9)	20 (9.9)	—		1.74 (0.992)	
Q21-Have you witnessed another nurse using a smartphone during work?	PI	5 (2.2)	22 (9.5)	69 (29.7)	104 (44.8)	31 (13.4)	0.030	3.56 (0.942)	0.060
	PII	12 (5.9)	31 (15.3)	62 (30.5)	66 (32.5)	32 (15.8)		3.37 (1.102)	

Item		Strongly disagree	Disagree	Uncertain	Agree	Strongly agree	*p* ^a^	Mean (SD)	*p* ^b^
Opinion about Smartphone restriction policies									
Q22-I do not want students to use their smartphone during clinical practice.	PI	34 (14.6)	61 (26.2)	71 (30.5)	52 (22.3)	15 (6.4)	0.002	2.80 (1.136)	0.094
	PII	56 (27.7)	35 (17.3)	61 (30.2)	31 (15.3)	19 (9.4)		2.61 (1.293)	
Q23-I do not want nurses to use their smartphone during working hours.	PI	25 (10.7)	60 (25.8)	75 (32.2)	59 (25.3)	14 (6)	<0.001	2.90 (1.084)	0.003
	PII	52 (25.7)	40 (19.8)	66 (32.7)	30 (14.9)	14 (6.9)		2.57 (1.216)	
Q24-There is a need for policies restricting smartphone use by students during clinical practice.	PI	35 (15)	69 (29.6)	77 (33)	50 (21.5)	2 (0.9)	<0.001	2.64 (1.009)	<0.001
	PII	62 (30.7)	59 (29.2)	44 (21.8)	29 (14.4)	8 (4)		2.32 (1.167)	
Q25-There is a need for policies restricting smartphone use by nurses during working hours.	PI	31 (13.3)	69 (29.6)	80 (34.3)	50 (21.5)	3 (1.3)	<0.001	2.68 (0.998)	<0.001
	PII	66 (32.7)	52 (25.7)	48 (23.8)	25 (12.4)	11 (5.4)		2.32 (1.205)	

P1 Period 1, P2 Period 2, SD standard deviation.

^a^ Chi-square statistics.

^b^ Mann–Whitney U statistics.

Use of smartphones for personal matters during practicum (Q12) showed a small but significant change between periods (*p* = 0.049), with a shift from “never” being the most frequent response in Period I toward a higher proportion of “rarely” responses in Period II; however, no significant differences in mean scores were observed (1.90 ± 0.96 vs. 1.85 ± 0.78; *p* = 0,937).

No significant differences were found for smartphone use in the presence of patients (Q13) or during training sessions (Q14), with very low frequencies reported in both periods.

Regarding distraction, no significant differences were observed in self-reported distraction due to smartphone use (Q15). In contrast, distraction due to other students’ smartphone use (Q16) decreased significantly in Period II (*p* = 0.034), with lower mean scores (1.28 ± 0.58 vs. 1.43 ± 0.74; *p* = 0.029), indicating a reduction in perceived distraction caused by peers.

No significant differences were found in witnessing other students (Q17) or nurses (Q18) being distracted by smartphone use. Similarly, observing tutors or reference nurses using smartphones in the presence of patients (Q19) or during training sessions (Q20) did not differ significantly between periods.

However, witnessing general smartphone use by nurses during work (Q21) showed a significant change in response distribution (*p* = 0.030), although mean differences were not statistically significant (3.56 ± 0,942 vs. 3.37 ± 1.102; *p* = 0.060). Specifically, responses in Period II shifted toward lower-frequency categories, with more students reporting “rarely” and “sometimes” compared to Period I, suggesting a shift toward less frequent but still present smartphone use among nurses rather than a substantial reduction in overall frequency.

Opinions on smartphone restriction policies changed significantly between periods. Agreement with restricting smartphone use by students (Q22, *p* = 0.002) and nurses (Q23, *p* < 0.001) decreased in Period II, with a significant reduction observed for restrictions on nurses based on mean scores (*p* = 0.003). Similarly, the perceived need for restriction policies for both students (Q24) and nurses (Q25) decreased significantly in Period II (both frequency distributions and mean scores, *p* < 0.001), indicating a shift toward less restrictive attitudes regarding smartphone use in clinical settings.

To further explore these patterns, Table 4 presents changes in smartphone use for personal matters during practicum and distraction caused by other students’ smartphone use across academic years between Period I and Period II.

**Table 4:** Changes regarding the mobile use for personal matters and distraction caused by other students’ use of their smartphone during practicum for each academic year between Period I and Period II.

Changes among academic years	Frequency *N* (%)												
	First year			Second year			Third year			Fourth year			Total
	Rarely	Frequently	*p* ^a^	Rarely	Frequently	*p* ^a^	Rarely	Frequently	*p* ^a^	Rarely	Frequently	*p* ^a^	*p* ^a^
PI-I have used my mobile for personal matters (e.g., calling, sending messages, accessing social networks, etc.) during the practicum (Q12)	29 (16.3)	6 (10.7)	0.526	63 (35.4)	8 (14.3)	0.063	49 (27.5)	22 (39.3)	0.015	37 (20.8)	20 (35.7)	0.293	0.005
PII-I have used my mobile for personal matters (e.g., calling, sending messages, accessing social networks, etc.) during the practicum (Q12)	30 (18.1)	4 (10.8)		55 (33.1)	15 (40.5)		46 (27.7)	6 (16.2)		35 (21.1)	12 (32.4)		0.197
	Mean	SD	*p* ^b^	Mean	SD	*p* ^b^	Mean	SD	*p* ^b^	Mean	SD	*p* ^b^	*p* ^c^
PI-Others’ distraction due to their use (Q17-Q18)	1.78	0.95	0.672	1.70	0.81	0.147	2.05	0.75	0.490	2.17	0.63	0.992	<0.001
PII-Others’ distraction due to their use (Q17-Q18)	1.57	0.61		1.85	0.76		2.00	0.90		2.20	0.82		0.006

Rarely (includes never and rarely) and frequently (sometimes, often and always).

PI Period 1; PII Period 2.

^a^ Chi-square statistics.

^b^ Mann–Whitney U statistics.

^c^ Kruskal-Wallis statistics.

In Period I, the frequency of personal smartphone use during practicum differed significantly across academic years (*p* = 0.005). Specifically, third and fourth-year students reported more frequent use (39.3 and 35.7%, respectively) compared to first and second-year students (10.7 and 14.3%, respectively). No significant differences were observed across academic years in Period II. When comparing periods within each academic year, a significant difference was observed only among third-year students, with a higher frequency of personal smartphone use reported in Period I (*p* = 0.015).

Regarding distraction caused by others’ smartphone use, no significant differences were found between periods within individual academic years. However, significant differences were observed across academic years in both periods (Period I: *p* < 0.001; Period II: *p* = 0.006), with higher levels of perceived distraction reported among students in more advanced academic years, particularly among fourth-year students.

The correlations between distraction experiences, opinions on smartphone restriction policies, smartphone professional and academic use, and perceived work efficiency for Period I and Period II are presented in Table 5.

**Table 5:** Correlations of distraction experiences with opinions about smartphone restriction policies, smartphone academic and professional use, and students’ perception of their work efficiency when using the smartphone for Period I and Period II.

Period	Distraction due to own use (Q15-Q16)		Distraction due to others’ use (Q17-Q18)	
	*r*	*p*	*r*	*p*
Period I				
Opinions about smartphone restriction policies (Q22-Q25)*	0.114	0.084	0.139	0.034
Smartphone professional use (Q1-Q5) *	−0.001	0.993		
Smartphone use for accessing university resources and sending documents for assessment (Q6-Q7) *	0.068	0.308		
Smartphone use makes work more efficient (Q10) *	0.012	0.86		
Distraction due to others’ use (Q17-Q18) *	0.510	<0.001		
Period II				
Opinions about smartphone restriction policies (Q22-Q25) *	0.041	0.566	0.061	0.390
Smartphone professional use (Q1-Q5) *	0.216	0.002		
Smartphone use for accessing university resources and sending documents for assessment (Q6-Q7) *	0.127	0.071		
Smartphone use makes work more efficient (Q10) *	0.016	0.822		
Distraction due to others’ use (Q17-Q18) *	0.487	<0.001		

*Spearman’s Rho statistics.

PI Period 1; PII Period 2.

In Period I, distraction due to students’ own smartphone use was not significantly correlated with opinions on restriction policies, professional smartphone use, academic use, or perceived work efficiency. However, a strong positive correlation was found between distraction due to own use and distraction due to others’ use (*r* = 0.510, *p* < 0.001), indicating that students who reported higher distraction from their own smartphone use also tended to perceive greater distraction caused by others. Regarding distraction due to others’ smartphone use, a weak but significant positive correlation was observed between this variable and opinions on restriction policies (*r* = 0.139, *p* = 0.034), suggesting that higher perceived distraction from others’ use was associated with greater support for restrictive policies.

In Period II, distraction due to students’ own smartphone use was significantly and positively correlated with professional smartphone use (*r* = 0.216, *p* = 0.002), while no significant associations were observed with opinions on restriction policies, academic use, or perceived work efficiency. This indicates that students reporting higher levels of professional smartphone use also tended to report greater distraction from their own use. Distraction due to others’ smartphone use was not significantly correlated with any of the studied variables, although it remained strongly correlated with distraction due to own use (*r* = 0.487, *p* < 0.001).

Table 6 shows changes in distraction experiences and opinions on smartphone restriction policies according to clinical practice setting (hospital vs. non-hospital) between Period I and Period II. No significant differences were observed between hospital and non-hospital settings in either period regarding distraction due to students’ own smartphone use or due to others’ use. Similarly, no significant changes between periods were found within each setting for these variables.

**Table 6:** Changes regarding distraction experiences and opinions about restriction policies according to students’ clinical practice setting between Period I and Period II.

Setting	Distraction due to own use (Q15-Q16)^a^				Distraction due to others’ use (Q17-Q18)^a^						Opinion on restriction policies (Q22–Q25)^b^				
	Mean (SD)				Mean (SD)						Mean (SD)				
	Period I	*p* ^a^	Period II	*p* ^a^	*p* ^b^	Period I	*p* ^a^	Period II	*p* ^a^	*p* ^b^	Period I	*p* ^a^	Period II	*p* ^a^	*p* ^b^
Hospital	1.36 (0.57)	0.664	1.24 (0.41)	0.256	0.177	2 (0.81)	0.146	1.92 (0.79)	0.752	0.428	2.73 (0.87)	0.562	2.44 (1.10)	0.395	0.003
Non-hospital	1.37 (0.56)		1.37 (0.57)		0.955	1.83 (0.76)		1.89 (0.84)		0.766	2.78 (0.85)		2.53 (1.05)		0.228

SD standard deviation; PI Period 1; PII Period 2.

p^a^ Mann–Whitney U statistics for students’ clinical practice setting (hospital vs. non-hospital) for Period I and for Period II separately.

p^b^ Mann–Whitney U statistics for students’ clinical practice setting (hospital vs. hospital and; non-hospital vs. non-hospital) between Period I and Period II.

^a^ The higher the value, the greater the frequency.

^b^ The higher the value, the greater the agreement.

Opinions on smartphone restriction policies differed significantly between periods in the hospital setting, with a significant decrease in agreement in Period II compared to Period I (2.44 ± 1.10 vs. 2.73 ± 0.87, *p* = 0.003), indicating a shift toward more permissive attitudes regarding smartphone use in hospital-based clinical placements. In contrast, in the non-hospital setting, no significant differences were observed, with opinions remaining stable across periods.

Additional analyses examining associations between smartphone use, perceived efficiency, distraction, and attitudes toward restriction policies are presented in the Supplementary Tables S1, S2.

Overall, consistent patterns were observed across both periods, indicating that more restrictive attitudes toward smartphone use were associated with lower levels of professional and academic smartphone use, as well as reduced perceived work efficiency. These relationships were moderate and statistically significant, although slightly attenuated in Period II, particularly for academic use.

Furthermore, positive associations were identified between academic smartphone use, professional use, and perceived efficiency, suggesting that students who reported greater engagement with smartphones for academic purposes also tended to use them more frequently in clinical contexts and perceived them as enhancing their work efficiency. These relationships were stronger in Period II, particularly between academic and professional use.

## 4 Discussion

The present study provides an updated overview of smartphone use among nursing students during clinical placements, including perceptions of smartphones as a tool for professional use and their potential to cause distraction, attitudes towards smartphone use policies, and changes observed over a five-year period. The results indicate a substantial evolution in smartphone use during clinical placements over time. While daily smartphone use patterns changed between the two study periods, the most notable finding was the increased integration of smartphones into both professional and academic activities during clinical practice.

Compared with the earlier cohort, students in Period II reported more frequent use of smartphones for communication with healthcare professionals, access to drug-related information, consultation of clinical resources, use of healthcare applications, and completion of academic tasks. These findings suggest that smartphones are increasingly perceived not only as personal communication devices but also as tools that support learning and clinical decision-making during placements. Consistent with previous research, health sciences students frequently use their personal smartphones to access healthcare-related information and educational resources in clinical settings (16–18).

Despite these changes, social media use remained highly prevalent in both study periods, with Instagram and TikTok emerging as dominant platforms in Period II. This finding suggests that the growing professional and academic use of smartphones has occurred alongside, rather than replacing, their social role among nursing students, a pattern also reported in previous studies (19, 20).

The observed changes in smartphone use suggest a progressive integration of mobile technologies into nursing students’ clinical learning experiences. Rather than functioning solely as communication tools, smartphones appear to support information retrieval and clinical decision-making processes during placements. Similar findings have been reported among healthcare students, who frequently use mobile applications and online resources to support clinical tasks and medication-related calculations (21). The observed increase in professional and academic smartphone use may reflect the growing digitalization of healthcare education and clinical practice, as well as the progressive integration of mobile technologies into clinical learning. Although (22) supported the educational and professional value of smartphones, our findings extend previous evidence by suggesting that this professional use has become more established over time within the clinical learning environment.

However, greater integration did not diminish students’ concerns or awareness of the challenges associated with smartphone use in clinical settings. Students continued to recognize that smartphone use could be perceived as potentially unprofessional or unsafe, with no significant differences between study periods. This finding highlights an apparent tension between the educational benefits of smartphones and concerns regarding professionalism, patient safety, and appropriate use in clinical environments. Previous studies have noted that personal smartphones may blur the boundaries between professional and personal activities, raising concerns about distraction, professional conduct, and confidentiality (11, 23). At the same time, healthcare systems are undergoing a rapid process of digital transformation, and mobile technologies are increasingly embedded within clinical workflows (24). Together, these developments underscore the need to balance the expanding role of mobile technologies in clinical practice with ongoing concerns regarding their appropriate and professional use.

Contrary to concerns that greater smartphone use may lead to increasing distraction in clinical settings, our findings showed no increase in self-reported distraction over the five-year period. Moreover, perceived distraction caused by other students’ smartphone use decreased significantly in Period II. This suggests that the growing integration of smartphones into clinical training has not necessarily been accompanied by a greater perception of disruption.

The strong correlation observed between distraction caused by students’ own smartphone use and distraction attributed to others in both study periods indicates that these experiences may be closely interconnected. Interestingly, in Period II, higher levels of professional smartphone use were associated with greater self-reported distraction. While this finding does not imply causality, it may reflect the increasing presence of smartphones within clinical activities and highlights the need to promote digital professionalism and appropriate smartphone use during clinical placements. Previous studies have reported concerns regarding excessive smartphone use, distraction, procedural errors, and potential risks to patient safety among healthcare students and professionals (10, 25).

Students in Period II expressed significantly less support for restrictive smartphone policies, particularly in hospital-based clinical placements. This finding is consistent with the observed increase in professional and academic smartphone use and may reflect students’ increasing recognition of the practical value of smartphones for learning and clinical activities. Notably, this shift towards more permissive attitudes occurred without a corresponding increase in perceived distraction, suggesting that students may better distinguish between appropriate professional use and inappropriate personal use of smartphones in clinical environments.

Although achieving an appropriate balance between technological innovation and patient safety remains challenging (10), healthcare and educational institutions should continue promoting clear guidance on digital professionalism, responsible smartphone use, confidentiality and patient-centred care (26). Within this context, the challenge may not be whether smartphones should be used during clinical training, but rather how nursing students can be supported in using these technologies responsibly, professionally, and in ways that protect patient safety and privacy. Strengthening digital professionalism competencies—including ethical decision-making, accountability, digital literacy and appropriate online conduct—may represent a key strategy to achieve this balance (27).

These findings further support the importance of digital professionalism as a core competency for future nurses. The more permissive attitudes towards smartphone use observed in Period II may also reflect broader changes in clinical culture. During clinical placements, nursing students are exposed to the behaviours and practices of healthcare professionals, who often serve as important role models in shaping professional norms and expectations (28). As smartphones become increasingly embedded in everyday clinical workflows, students may perceive their use as a legitimate component of contemporary healthcare practice. However, the normalization of smartphone use should not diminish awareness of the potential risks associated with distraction, confidentiality breaches, infection control, and patient safety (9, 10, 23). Consistent with previous research, these findings highlight the importance of clear institutional guidance and professional role modelling to ensure that smartphones are used appropriately and responsibly within clinical environments (1, 12).

The growing integration of smartphones into clinical education may become even more relevant as mobile access to artificial intelligence-based educational and clinical support tools becomes increasingly common among nursing students. While these technologies offer important opportunities to enhance learning and clinical decision-making, they also reinforce the need to strengthen digital professionalism competencies, including critical appraisal of information, ethical decision-making, accountability, and the responsible use of technology in patient care (27, 29).

This study has several limitations that should be considered when interpreting the findings. First, the study was conducted in a single nursing school in Spain, which may limit the generalizability of the results to other educational institutions, healthcare systems, and cultural contexts. Second, participants were recruited using a convenience sampling strategy and although nearly 60% of the target population participated, selection bias cannot be ruled out. Third, all variables were measured through self-reported questionnaires and may therefore be subject to recall bias and social desirability bias, particularly regarding potentially inappropriate smartphone use during clinical placements. Moreover, the study did not assess the quality, validity, currency, or reliability of the information or digital resources accessed via smartphones. Therefore, the findings do not allow conclusions to be drawn regarding the quality of the resources consulted by students. Fourth, comparisons between Period I and Period II were based on two independent cohorts rather than repeated assessments of the same individuals; therefore, observed differences may reflect cohort effects or broader changes in educational, technological and clinical environments over time. Finally, data collection procedures differed between study periods, with paper-based questionnaires used in Period I and online questionnaires used in Period II. Although the same instrument was employed in both periods, differences in survey administration may have influenced response patterns and should be considered when interpreting comparisons across cohorts.

## 5 Conclusion

This study identified important changes in smartphone use among nursing students during clinical placements over a five-year period. Compared with the earlier cohort, students reported greater professional and academic use of smartphones, particularly for accessing clinical information, supporting decision-making, communicating with healthcare teams, and completing educational activities.

Despite this increased use, smartphone-related distraction did not increase and perceptions of distraction caused by peers decreased. At the same time, students expressed less support for restrictive smartphone policies, suggesting a growing acceptance of smartphones as legitimate educational and clinical tools.

Taken together, these findings indicate an ongoing normalization of smartphone use within nursing clinical education. Rather than supporting broad prohibitive approaches, the results suggest that educational institutions and healthcare organizations should focus on promoting digital professionalism, responsible smartphone use, and clear guidance regarding confidentiality, patient safety, and professional conduct.

Future research should explore these issues across diverse educational and clinical settings, including the quality and reliability of the digital resources accessed by nursing students, assess the impact of emerging technologies such as artificial intelligence-enabled mobile tools, and evaluate strategies designed to strengthen digital professionalism competencies among nursing students and healthcare professionals.

## Data Availability

The raw data supporting the conclusions of this article will be made available by the authors, without undue reservation.
