# Supplementary material for: Smartphone use among nursing students during clinical placements: a five-year comparison of use, distraction, and regulatory perceptions
Source: Front Public Health. 2026 Oct 8;14:1987602. doi: 10.3389/fpubh.2026.1987602 (PMC13687057; doi:10.3389/fpubh.2026.1987602)
Supplement: Supplementary file 1 [file Supplementary_file_1.DOCX]

**Supplementary material**

**Table S1.** Correlations between opinions on smartphone restriction policies, smartphone professional and academic use, and perceived work efficiency in Period I and Period II

|  | Opinions about smartphone restriction policies (Q22-Q25) | |
| --- | --- | --- |
|  | **r** | **p** |
| Period I |  |  |
| Smartphone professional use (Q1-Q5) * | - 0.296 | <0.001 |
| Smartphone use for accessing university resources and sending documents for assessment (Q6-Q7) * | - 0.236 | <0.001 |
| Smartphone use makes work more efficient (Q10) * | - 0.351 | <0.001 |
| Period II |  |  |
| Smartphone professional use (Q1-Q5) * | - 0.215 | 0.002 |
| Smartphone use for accessing university resources and sending documents for assessment (Q6-Q7) * |  |  |
| Smartphone use makes work more efficient (Q10) * | - 0.351 | <0.001 |

* Spearman’s Rho statistics

Abbreviations: PI Period 1; PII Period 2

**Table S2.** Correlations between smartphone professional use, academic use, and perceived work efficiency in Period I and Period II

|  | Smartphone professional use (Q1-Q5) | | Smartphone use makes work more efficient (Q10) | |
| --- | --- | --- | --- | --- |
|  | **r** | **p** | **r** | **p** |
| Period I |  |  |  |  |
| Smartphone use for accessing university resources and sending documents for assessment (Q6-Q7) * | 0.482 | <0.001 | 0.320 | <0.001 |
| Smartphone use makes work more efficient (Q10) * | 0.320 | <0.001 |  |  |
| Period II |  |  |  |  |
| Smartphone use for accessing university resources and sending documents for assessment (Q6-Q7) * | 0.690 | <0.001 | 0.275 | <0.001 |
| Smartphone use makes work more efficient (Q10) * | 0.329 | <0.001 |  |  |

* Spearman’s Rho statistics

Abbreviations: PI Period 1; PII Period 2
